# The Reconnecting the Hand and Arm with Brain (ReHAB) Commentary on “An Integrated Brain-Machine Interface Platform With Thousands of Channels”

**DOI:** 10.2196/16339

**Published:** 2019-10-31

**Authors:** Robert F Kirsch, A Bolu Ajiboye, Jonathan P Miller

**Affiliations:** 1 VA Rehabilitation Research and Development Service, Center for Functional Electrical Stimulation Case Western Reserve University Cleveland, OH United States; 2 Case Western Reserve University University Hospitals Cleveland Medical Center Cleveland, OH United States

**Keywords:** brain computer interfacing, intracortical recording, neural engineering, neurosurgery

## Abstract

Intracortical brain-machine interfaces are a promising technology for allowing people with chronic and severe neurological disorders that resulted in loss of function to potentially regain those functions through neuroprosthetic devices. The penetrating microelectrode arrays used in almost all previous studies of intracortical brain-machine interfaces in people had a limited recording life (potentially due to issues with long-term biocompatibility), as well as a limited number of recording electrodes with limited distribution in the brain. Significant advances are required in this array interface to deal with the issues of long-term biocompatibility and lack of distributed recordings. The Musk and Neuralink manuscript proposes a novel and potentially disruptive approach to advancing the brain-electrode interface technology, with the potential of addressing many of these hurdles. Our commentary addresses the potential advantages of the proposed approach, as well as the remaining challenges to be addressed.

Over the past two decades, several academic research laboratories have advanced the science and implementation of intracortical brain-machine interfaces from exclusive use in nonhuman primates to investigative use in human volunteers with chronic neurological impairments under pilot clinical trials approved through the US Food and Drug Administration (FDA). Researchers in these labs have successfully demonstrated that these persons can use intracortical brain-machine interfaces to command movements of a computer cursor, a robotic arm, their own arm reanimated through functional electrical stimulation, and recently speech articulation. Our own group has developed a system that uses an intracortical brain-machine interface to command a functional electrical stimulation system that coordinates activation of paralyzed upper limb muscles to restore useful function. Our current study is the Reconnecting the Hand and Arm with Brain (ReHAB) clinical trial, which uses Blackrock recording and stimulation arrays.

As noted in the paper by Musk and Neuralink [1], the sampling of neurons by electrode arrays currently available for human applications is a tiny percentage of the relevant neural population. Access has been largely limited to cortical surfaces 1-2 millimeters deep and on gyri and not in sulci, which is where several key areas in the human brain are located. The performance of existing human grade electrodes deteriorates over a shorter time frame than would be acceptable for many clinical applications. Transmitting the recorded neural signals to the external world with high fidelity has relied on percutaneous connectors that are unlikely to provide permanent clinical solutions, or on low channel–count telemetry devices that are unlikely to provide adequate information for many applications. In addition to recording, intracortical stimulation through these same or similar electrodes is being tested to inject information into brain structures (eg, to restore sensation in paralyzed individuals). Intracortical stimulation applications face many of the same issues described above for recording but bring additional safety issues. The Musk and Neuralink paper hints at future brain stimulation capabilities, but these are not described and will not be discussed further in our commentary.

Based on our experiences, any significant step forward in human brain recording will require electrode technologies that provide orders of magnitude more information. This information will be derived from: (1) a much higher number of recording/stimulation contacts; (2) recording both surface and deep brain structures (eg, in sulci or noncortical regions), as well as from multiple brain areas with different functionality; and (3) different signal types (eg, single units and local field potentials). These electrodes should be able to be inserted safely and within a reasonable surgical time window, and should be well tolerated by the brain (ie, cause negligible damage upon insertion and be virtually invisible to the immune system to thus provide stable performance over decades). Hardware that records signals from these electrodes should extract relevant neural information from the high numbers of channels with high fidelity and in real-time, avoid percutaneous interfaces, and always be available for use.

The authors of this paper described a novel approach involving ultrafine polymer threads, each containing a dense linear array of 32 high-impedance electrodes, that are individually implanted into the cortex using a robotic device. The implantation robot can be controlled intraoperatively with micron precision to avoid small surface blood vessels and is able to implant up to six threads (192 electrodes) per minute. A small (23x18.5 millimeter) custom printed circuit board with onboard power is able to connect to up to 96 of the threads for a total of 3072 electrodes per implanted array, and digitized high-bandwidth neural data is streamed from the device using a single USB-C cable. Two different versions of the system were developed, one maximizing reliable manufacturing (using half as many leads) and another maximizing channel count. Testing in an awake rat demonstrated the ability to record signals interpreted as neural spike data from 43% of the channels. The authors conclude that this strategy may revolutionize brain-machine interfaces by recording from an unprecedented number of neurons.

Clinical application of brain-machine interfaces may require a very high channel count to allow recording from many neurons, so the relatively small number of contacts available using currently available platforms has hindered its development. The technology and processes presented in the Musk and Neuralink paper can certainly increase the channel count by an order of magnitude, providing a more detailed sampling of relevant signals as well as some welcome redundancy. Perhaps even more attractive about the proposed approach is the possibility of placing electrodes into areas of the brain that have been difficult or impossible to reach with existing intracortical arrays (eg, on the medical surfaces of sulci and subcortical structures). These new locations could potentially provide different types of information for enhancing brain-machine interface performance (eg, abstract planning of activities [including movement goals and sequencing], anticipated reward signals, and decision making), as well as better elucidate the interactions between different brain structures (eg, between different motor areas, processing of sensory information, and integration of sensory-motor activities). Such information is likely to be critical for successful performance in more complex brain-machine interface applications, such as multidimensional arm movement involving complex physical dynamics.

The use of individually implanted threads of electrodes is a clever way to allow an exponential increase of channel count over existing brain-machine interface technology, and the authors’ approach is well conceived with a good consideration of both physical and technical characteristics. However, the potential clinical application of this strategy is unclear since it has only been tested in a small number of rodents, with no comparison to existing approaches or verification of safety using histological analysis after implantation. The authors claim that their implants will have greater longevity than other options because of less immune response related to electrode stiffness and microvascular disruption, but no evidence is presented to support either of these assumptions, and improved durability was not verified using long-term implantation. It is not clear that blood vessels below the surface can be avoided, potentially critical for immune responses. The paper does not address the use of the thread electrodes for larger brains with more complex cortical structures (eg, the deeply folded structure of the human brain). The potentially implantable recording system as presented does not include hermetic sealing, a relevant power source (eg, battery, induction, or optical), or a technique (eg, wireless) for transmitting high bandwidth data out of the body without a percutaneous interface. Furthermore, the potential impact of noise and artifact has not been unequivocally established: It is uncertain whether the signals recorded in the rodent study actually represent meaningful neural data since the measured impedance was relatively low compared with penetrating electrodes typically used to measure single-unit neural activity, similar signals were seen on many adjacent channels, and no attempt was made to validate that the spike data had physiological characteristics typical of neural spiking patterns in the regions that were implanted. The technology is very innovative, but better validation will be necessary to establish its clinical potential.

Overall, this new technology is exciting because it attempts to directly address a number of bottlenecks that have hindered true clinical translation of intracortical arrays for use in brain-machine interfaces. The amount of high-resolution information that could be simultaneously recorded from cortical neurons may lead to new advances in the application of data mining and machine learning approaches to better understand cortical electrophysiological activity at the macro-, meso-, and microscale levels. Ultimately these multi-prong advances will likely result in advances in brain-controlled neuroprosthetics for addressing impaired function in neurologically compromised individuals. However, a responsible stance of cautious optimism must be taken. There is a long road between showing single neuron recordings in a handful of rodents to clinically translated human use, including proof (not just potential) of longevity, efficacy, and safety through an FDA-approved human clinical trial. If successful, the proposed technology (and future derived technologies) could pave the way for more widespread translation of intracortical brain-machine interfaces for medical applications in people with chronic neurological impairments.

## Abbreviations

FDA: US Food and Drug Administration
ReHAB: Reconnecting the Hand and Arm with Brain
